# Evaluation of Bone Healing Using Contrast-Enhanced Ultrasonography in Non-Operative Treatment of Tibial Fracture in a Puppy Dog

**DOI:** 10.3390/ani11020284

**Published:** 2021-01-23

**Authors:** Francesco Macrì, Vito Angileri, Teresa Russo, Maria Tomiko Russo, Marco Tabbì, Simona Di Pietro

**Affiliations:** 1 Department of Veterinary Sciences, University of Messina, Viale Palatucci, 98168 Messina, Italy; dpimedvet@gmail.com (F.M.); marcotb93@hotmail.it (M.T.); 2 Veterinary Practitioner, 91025 Marsala, Italy; vitoang@gmail.com; 3 Veterinary Practitioner, 98077 Santo Stefano di Camastra, Italy; teresarusso.vet@gmail.com; 4 Veterinary Practitioner, 95030 Pedara, Italy; mariarusso4@yahoo.com

**Keywords:** dog, tibial fracture, contrast-enhanced ultrasonography

## Abstract

**Simple Summary:**

This report describes the clinical presentation of a tibial fracture in a young dog. treated with a conservative approach and subjected to X-ray, B-mode, Color Doppler and contrast-enhanced ultrasound (CEUS) examinations during the bone healing, in order to assess hemodynamic changes during fracture healing. This report showed the application of CEUS in controlling the fracture healing process.

**Abstract:**

A 10-month-old mixed-breed male dog was presented with an oblique tibial fracture. The dog was treated with a Robert Jones-like bandage as a conservative approach, and was subjected to X-ray, B-mode, Color Doppler and contrast-enhanced ultrasound (CEUS) examinations during the fracture healing, in order to assess bone hemodynamic changes. B-mode, Power Doppler and CEUS examinations of the fracture gap were performed at 7, 20, 35, and 50 days post-trauma. Quantitative analysis of CEUS and perfusion parameters were obtained. On CEUS, a steep incline in signal numbers was visible in fracture gap at 7 days with peaks at 35 days, after which the vascularization decreases gradually over the next days. In this study, CEUS provided important information on the early stages of the callus formation and on the healing of neighboring tissues, allowing recognition of a correct bone healing. Moreover, the number of vascular signals on CEUS was greater than that on Doppler images on the same day. This report showed the application of CEUS in controlling the fracture healing process. CEUS could be a method of monitoring the remedial processes, assessing the tibial fracture perfusion characterized by low-velocity, small-volume blood flows.

## 1. Introduction

Fracture of the long bone is a commonly-encountered orthopedic problem in puppy dogs. Early diagnosis and correct classification are very important to choice the treatment. In routine practice, bone regeneration is evaluated employing several clinical and radiologic criteria such as the absence of pain on weight-bearing and periosteal callus observed on radiograph or computed tomography, which however appears after several weeks [1,2].

Recently, further diagnostic techniques that permit a more accurate control of the bone consolidation were studied. Ultrasonography was reported as a technique that can evaluate complete fracture healing earlier than conventional radiography in models of animals’ fractures [2,3,4,5].

Blood perfusion of the fracture gap and surrounding soft tissues is critical to bone healing and influences the treatment choice. In first week after bone fractures, the capillaries adjacent to the fracture site point out signs of neoangiogenesis [6]. Various factors can lead to failed fracture healing, causing an insufficient perfusion as diabetes and NSAIDs, or a hyperperfusion as infection [7].

In the last years, studies in human medicine have suggested that the contrast-enhanced ultrasound (CEUS), through gas-filled microbubbles evaluates tissues perfusion and appears useful for the evaluation of bone fracture and surrounding soft tissue microcirculation. Beyond its utility for basic research, CEUS can be also applied in clinical practice for the early detection of neoangiogenesis during the fibrocartilaginous callus formation [1,7,8]. The time-intensity curves parameters, generated during the quantitative analysis of CEUS, has been applied in various musculoskeletal indications, such as in rheumatoid arthritis [9] and for assessment of the post-operative perfusion of muscles and long bones non-union [10,11,12]. Some authors assessed the technical feasibility of CEUS in controlling non-infected long bone and non-union healing [13]. Furthermore, other authors intended to validate the diagnostic quality of CEUS as useful method for the pre-operative diagnosis of infected lower limb non-unions [14]. Compared to standard Power Doppler sonography, CEUS permits to evaluate also microvessel perfusion (2–7 nm), being more accurate [15].

In veterinary medicine, only one experimental study performed in a canine tibial osteotomy model showed the applicability of CEUS to evaluate fracture healing. The authors hypothesized that the perfusion of soft tissues around the fracture could be an early indicator of hemodynamic changes during bone healing. They showed that the neovascularization and blood perfusion during fracture healing was detected earlier in CEUS comparing with Doppler ultrasonography [2].

This report describes a case of tibial fracture in a puppy dog subjected to CEUS for assessing the neovascularization during fracture healing, by the analysis of vascular signal changes.

## 2. Materials and Methods

### 2.1. Ethical Statement

No ethical approval was required in compliance with European Directive 2010/63/UE and Italian Regulation D.Lgs n. 26/2014 because all the data derives from routine veterinary clinical practices. The use of contrast agent complied with the Veterinary Sciences Department’s Animal Ethics Council approval (protocol number (13/2017). Informed consent was obtained from dog owner before its inclusion in the study.

### 2.2. Clinical Examination

A 10-month-old mixed-breed male dog weighing 9 kg was admitted to the emergency room of the Veterinary Teaching Hospital of University of Messina for a suspected collision trauma.

A careful clinical examination was performed and systemic blood pressure measurement, complete blood count, and serum biochemistry (blood urea, creatinine, creatinine, glutamate pyruvate transferase, gamma glutamyl transferase, aspartate aminotransferase, and total protein). The orthopedic exam revealed a swelling in the right tibia, with crackling of the region at palpation. The dog showed severe pain and a lameness at the right hind limb.

The therapeutic choice, based on the young age of the subject, grade of lameness, and radiographic appearance of fracture, consisted of a Robert Jones-like bandage as a conservative approach, instead a surgical approach. The entire right hind limb was bandaged in order to stabilize the fracture site and promote biological healing.

In addition, 1 mg/Kg robenacoxib (Onsior^®^, Elanco S.p.a., Sesto Fiorentino, Italy) SID for 15 days SC, 8.75 mg/Kg amoxicillin/clavulanic acid (Synulox^®^, Pfizer Italia Srl, Latina, Italy) SID for 15 days SC and 2 mg/kg tramadol (Altadol^®^, Formevet s.r.l., Milano, Italy), SID for 5 days IM were administered.

Radiographic, ultrasound and CEUS examinations were performed to monitoring the fracture healing.

### 2.3. X-ray Procedure

The radiographic examination of the tibia was performed using an Analogic Radiographic/Fluoroscopic Table System (Dedalus Mb 90/20 IMX-2A, Imago Radiology S.r.l., Abbiategrasso (MI), 20081, Italy) with a digital radiography system (Fujifilm Medical Systems, Italy). Two orthogonal projections (medium–lateral and cranio–caudal) was performed in sedated dog at time of arrival in hospital (T0); the subsequent X-ray exams were performed on not sedated dog at 20 (T20), 35 (T35), 50 (T50) days after the first presentation.

### 2.4. B-Mode, Color Doppler and Contrast-Enhanced Ultrasound Procedures

B-mode, Power Doppler and CEUS examinations of the fracture gap were performed at 7 (T7), 20 (T20), 35 (T35), and 50 (T50) days after the trauma. The same investigator (FM) performed ultrasound examination, using a Mindray M9 ultrasound machine (Mindray Medical, Milan, Italy) equipped with a linear array broadband transducer (10 to 12 MHz). Dog was gently restrained without sedation. After detecting the lesions with standard ultrasound, CEUS was performed using a 5–7.5 MHz linear transducer with contrast agent capability. During CEUS a mechanical index of 0.06, 3 cm depth, 15 Hz pulse repetition frequency, and a 50% gain were set.

The contrast agent used was a sulfur hexafluoride signal enhancer (SonoVue^®^, Bracco Imaging, Milan, Italy), and it was prepared following the manufacturer’s recommendations. An aliquot (0.04 mL/kg of body weight) of the contrast medium was injected into the cephalic vein via three-way stopcock and a 20 G intravenous catheter, immediately followed by the rapid injection of 5 mL of 0.9% saline solution by a second operator (SD), in accordance with a methodology previously reported [16,17]. The dog received two bolus injections of contrast agent with at least 10 min interval between inoculations. The activation of a timer was performed simultaneously with the contrast agent dose inoculation. Care was taken to keep the probe in the same position for at least 2 min. Raw data (good-quality video clips for approximately 2 min) obtained during the second contrast enhanced examination were digitally stored on a hard disk and subsequently they were analyzed by the same operator.

Post processing quantitative analysis of video-clips was performed using the integrated specialized software contrast imaging quantitative analysis (QA) of the ultrasound machine (Mindray Medical, Milan, Italy), without movie clips exportation. One region of interest (ROI) of 0.3 cm^2^ area was manually drawn over the surrounding soft tissue as close as possible to the fracture gap and the periosteal callus. For the ROI, the following quantitative parameters were calculated from a time–intensity curve (TIC): The arrival time (AT): Time point where contrast intensity appears; peak intensity (PI): Contrast peak intensity; time to peak (TTP): The time when the contrast intensity reaches peak value, and area under curve (AUC): To calculate the area under the TIC during wash-in and wash-out.

## 3. Results

### 3.1. Radiographic Examinations

**Figure 1:**
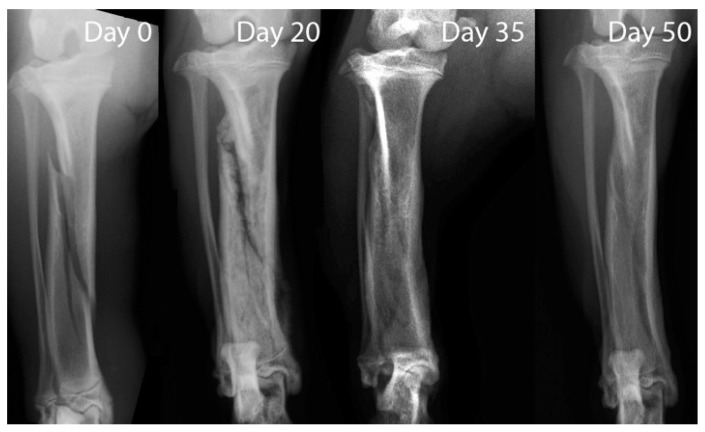
X-ray exam. The oblique fracture line of the tibial bone with intact fibula was visible on radiography at 0 day. Resorption of the fracture line was seen at day 20. At day 35, an early radiopaque callus was visible. Complete union of the fracture was obtained at day 50.

The radiographic study at T0 highlighted a tibial oblique non-displaced fracture; the fibula was intact. The X-ray exam at T20 showed irregular and blunted fracture margins due to the bone resorption. At T35 an early radiopaque callus with undulating and irregular contour was visible. The radiographic study at T50 showed an achieved complete osseous union of the fracture (Figure 1).

### 3.2. Ultrasonographic Examinations

The ultrasound study performed at T7 showed a discontinuity of the bone surface in the fracture gap that was filled with homogeneous hypoechoic to anechoic areas, due to the hematoma formation around the fracture gap. No Doppler signal was visible.

At T20 the ultrasound examination showed uneven and rounded fracture borders due to bone resorption; the soft tissue was heterogeneous and represented the mature hematoma. Poor number of Doppler signals were visible.

**Figure 2:**
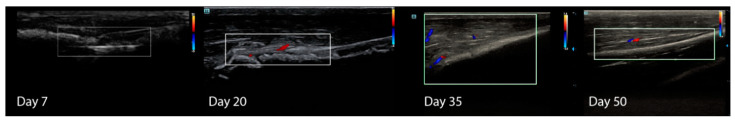
Doppler ultrasonography of the tibial bone healing: an increase in the number of vascular signals was seen at days 35, after which the vascularization decreased gradually.

At T35 B-mode ultrasound showed the presence of hyperchoic, regular, and homogeneous fibro-cartilaginous tissue into the fracture site due to formation of the callus; the fracture lines were almost completely disappeared. An increase of the number of signals was visible at the callus surface. Finally, the ultrasound examination at T50 showed a thin continuous line of the periosteal bone, demonstrating the successful calcification with the absence of newly formed tissue around the bone. The Doppler examination hardly revealed a very low number of vessel signals (Figure 2).

**Figure 3:**
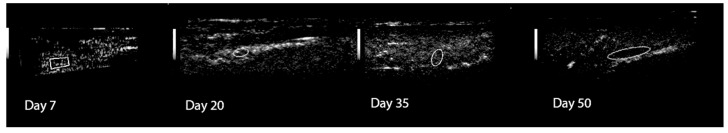
Peak intensity after contrast agent injection on contrast-enhanced ultrasound (CEUS) of the tibia during the bone healing. At 35 day the contrast agent shows the maximum enhancement. Region of interest (ROI) was manually drawn over the surrounding soft tissue as close as possible to the fracture gap.

On contrast-enhanced ultrasonography, a steep incline in signal numbers is visible after the first week, with peaks at day 35, after which the vascularization decreases gradually over the next days. The qualitative CEUS examination at T7 revealed poor vascular signals from surrounding soft tissue after contrast agent injection; at T20 vascular signals in the areas adjacent to the fracture were increased, as well as in the fracture gap, whereas at T35 a marked increase of the vascular network was detected. The vascularization decreased gradually until 50 days after the trauma, as demonstrated by low vascular signals (Figure 3).

**Figure 4:**
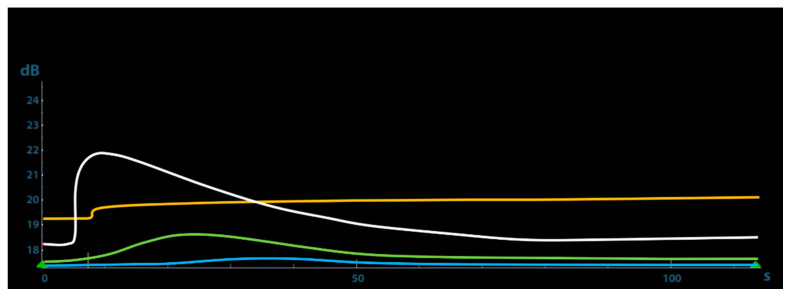
Time–intensity curves (TICs) of the contrast agent inflow into the fracture gap of the dog during the bone healing. Light blue line is TIC at 7 day; green line is TIC at 20 day; white line is TIC at 35 day (see the maximum of contrast agent inflow) and yellow line is TIC at 50 day.

The quantitative CEUS at T7 showed a very slow wash-in time, as demonstrated on TIC by the long AT and a very low PI. At T20 the TIC showed a faster wash-in and a short AT, whereas at T35 a very rapid wash-in with a low AT and a very high PI. This parameter peaked 21.87 dB at 35 days after trauma and then gradually decreased (Figure 4). Table 1 shows the results of the quantitative CEUS parameters recorded during the study.

## 4. Discussion

This report demonstrated the usefulness of CEUS in controlling the fracture healing process by the evaluation of hemodynamic changes during bone healing in a puppy dog affected by a tibial fracture.

The ultrasound contrast medium, persisting in the blood vascular system, allowed the real-time visualization and analysis of the qualitative and quantitative blood perfusion and neovascularization during tibial fracture healing.

Due to its limited soft tissue coverage and the wide risk of contamination in open fractures, the tibia is quite susceptible to not regenerating. In this case, CEUS provided important information on the early stages of the callus formation and on the healing of neighboring tissues, allowing recognition of a correct bone healing.

Contrast-enhanced results of the present report after one week indicated the presence of poor vascular signals from the surrounding soft tissue of the fracture site. After 20 days from the trauma, CEUS showed a moderate and slow periosteal callus enhancement that becomes rapid and reaches its peak at 35 days, highlighting the high tissue vascularization due to local angiogenesis and then decreases gradually over the next days, according to a normal fracture healing process.

In this case, complete healing was determined by CEUS much faster than reported in an adult dog model [2], 132 vs. 50 days, confirming that puppies have an excellent capacity for regeneration of tissues and bone. In these patients, the periosteum is thick and richly vascular. These factors make the capacity for osteogenesis excellent in most cases, allowing a rapid bone healing [18,19,20].

In this report, vascular signals from surrounding soft tissue of the fracture were detected on CEUS at 7 days after trauma, whereas Doppler ultrasonography could not detect any signal. Moreover, the number of vascular signals on CEUS was greater than that on Doppler images on the same day. Since CEUS is an imaging technique in which the backscattered contrast-enhanced signals from microbubbles are much greater than the other signals at the appropriate harmonic wavelength [2], it permits to detect a small vascular flow, which can be inconspicuous on Doppler ultrasonography [21,22,23].

The opportunity of detecting pathological perfusion pattern during the bone healing is an essential aspect in traumatology to correct the therapeutic choice. Further studies are indicated to assess perfusion in bone regeneration comparing different surgical approaches.

## 5. Conclusions

We have presented a clinical application of CEUS in monitoring the bone healing during a tibial fracture in a young dog CEUS resulted in promising method of monitoring the remedial processes, assessing the tibial fracture perfusion characterized by low-velocity, small-volume blood flows. However, this result requires further observation in a larger population animal study.

CEUS can be suitable as non-invasive diagnostic technique complementing conventional modalities of fracture and non-union diagnostics.

Furthermore, CEUS is an effective method in time and cost that allows the evaluation of contrast distribution in tissues in real time, completely avoiding radiation.

## Figures and Tables

**Table 1:** Quantitative contrast-enhanced ultrasound (CEUS) parameters in relations to ROI area.

Parameters	T7	T20	T35	T50
AT (sec)	20	7	6.20	10
TTP (sec)	34.48	22.60	11.07	13.39
PI (dB)	17.5	18.50	21.87	19.69
AUC	348.61	1461.24	2095.52	1845.64

ROI—region of interests; AT—arrival time; TTP—time to peak; PI—peak intensity; AUC—area under curve.

## Data Availability

The data presented in this study are available on request from the corresponding author.
